# Promoting fit bodies, healthy eating and physical activity among Indigenous Australian men: a study protocol

**DOI:** 10.1186/1471-2458-12-28

**Published:** 2012-01-11

**Authors:** Lina A Ricciardelli, David Mellor, Marita P McCabe, Alexander J Mussap, David J Hallford, Matthew Tyler

**Affiliations:** 1 School of Psychology, Deakin University, 221 Burwood Hwy, Burwood VIC 3125, Australia

## Abstract

**Background:**

Overall the physical health of Indigenous men is among the worst in Australia. Research has indicated that modifiable lifestyle factors, such as poor nutrition and physical inactivity, appear to contribute strongly to these poor health conditions. To effectively develop and implement strategies to improve the health of Australia's Indigenous peoples, a greater understanding is needed of how Indigenous men perceive health, and how they view and care for their bodies. Further, a more systematic understanding of how sociocultural factors affect their health attitudes and behaviours is needed. This article presents the study protocol of a community-based investigation into the factors surrounding the health and body image of Indigenous Australian men.

**Methods and design:**

The study will be conducted in a collaborative manner with Indigenous Australian men using a participatory action research framework. Men will be recruited from three locations around Australia (metropolitan, regional, and rural) and interviewed to understand their experiences and perspectives on a number of issues related to health and health behaviour. The information that is collected will be analysed using modified grounded theory and thematic analysis. The results will then be used to develop and implement community events in each location to provide feedback on the findings to the community, promote health enhancing strategies, and determine future action and collaboration.

**Discussion:**

This study will explore both risk and protective factors that affect the health of Indigenous Australian men. This knowledge will be disseminated to the wider Indigenous community and can be used to inform future health promotion strategies. The expected outcome of this study is therefore an increased understanding of health and health change in Indigenous Australian men, the development of strategies that promote healthy eating and positive patterns of physical activity and, in the longer term, more effective and culturally-appropriate interventions to improve health.

## Background

Overall, the physical health of Indigenous men is among the worst in Australia, and very poor in comparison to other first nations [1,2]. Recent reports from the Australian Bureau of Statistics [3] have indicated that the life expectancy for Indigenous Australian men is 11.5 years lower than that of non-Indigenous men. Further, their mortality rates are at least two times higher than that of non-Indigenous Australians, and at least four to five times higher in young adult and middle-aged groups [3]. The most significant conditions contributing to the burden of disease amongst Indigenous Australian men are cardiovascular disease, chronic respiratory disease, diabetes, and obesity [4]. This adversity is also reflected in their poor mental health [1,5] and negative body esteem [6-8].

A major factor contributing to Indigenous Australian's physical and emotional health is the significant lifestyle changes that have taken place since colonisation [9]. These include an adoption of "Westernized" diets, increased consumption of foods high in fat, sugar and salt, and a decline of physical activity. This is a marked contrast to the Indigenous lifestyle before European colonisation where Aboriginal people "gathered, trapped and hunted indigenous foods that were predominantly low in dietary energy, fat, salt and sugar, and high in fibre and complex carbohydrates. They also expended considerable physical energy in obtaining sufficient water and food to survive" [[9], p. 16]. Indeed, two of the major contributors to the current condition of Indigenous Australians health are poor nutrition [10,11] and sedentary behaviour [12]. For example, findings from the Heart Health Project have indicated that that Indigenous Australians are not eating fruit and vegetables or exercising at levels recommended in the dietary guidelines for Australians [13]. Other studies have also indicated that the main risk factors contributing to the disease burden of Indigenous Australians, and the pronounced health gap between Indigenous and non-Indigenous Australians [14] are modifiable factors including physical inactivity, high blood cholesterol, and low fruit and vegetable intake [15].

Another significant factor to consider is that many Aboriginal people have had and continue to have little or no control over their circumstances. As argued by Palmer [[16], p. 23]: "It's hard when people push you in all sorts of different directions; one group wants you to modernise, the other wants you to stay traditional. What do Central Australian Aboriginal men want? This is what our people should be thinking about-not what others think is best for us! ... Aboriginal men need to have a purpose in daily life and our culture; it is both our Tjurunga^1 ^and family that really matter. As Aboriginal men we are supposed to uphold the lore and take care of Tjurunga and family, then pass that knowledge to our sons. As men maybe then we will have a purpose again and make us stronger in men's lore."

The above discussion presents some of the risk factors that may contribute to the poor physical and mental health of Indigenous men. It also suggests that there may be protective factors that can be targeted to enhance their health. Despite the frequent recognition that sociocultural factors may play a crucial role in health outcomes for Indigenous Australians, these are often not examined because they are very difficult to measure [17,18]. To assist in developing and implementing more effective health change strategies an improved understanding of these factors is necessary. An array of these factors, either identified by previous research or raised through broader discourse around the health of Australia's Indigenous peoples, will now be considered.

### Indigenous men's understanding and value placed on health

Indigenous Australians do not place importance on their health in the same way as non-Indigenous Australians. In fact, many traditional Indigenous concepts of health are incongruent with Western views and those represented in current models of health psychology and allied disciplines that are used to develop health programs for Indigenous people [19-22]. As argued by Anderson [23], Indigenous people do not make the Platonic and Cartesian divide between mental and physical being; instead "health includes the physical, social, emotional, cultural and spiritual wellbeing ... and this is not only of the individual but of the whole community" [[24], p. 68]. McCoy [[25], p. 220] also points out that in some communities a "person is healthy (or *playa) *when their body (*yarnangu) *is in a right relationship with their inner spirit (*kurrun*) and with others (*walytja*)". Therefore, we need to better understand and incorporate these views in any new health initiatives [23].

### Fitness, health and body image

Similarly, Aboriginal men's criteria for physical esteem and their views of an ideal body are often far removed from the fit, lean and muscular male body epitomized in the Western media [26]. McDonald [21] has argued that "for indigenous people who have lived through periods of feast and famine, thin bodies were not valued". Moreover, many Indigenous people are of the view that "the ability to put on weight during good seasons enabled people to survive the bad seasons"; "thinness can indicate weakness, excessive worry, or ill-health"; and a skinny body is viewed as "sexually unattractive" [[21], p. 88].

In stark contrast to these traditional views, many men from non-Western cultural groups [27-30], including Indigenous Australian men [6], are increasingly exposed to media representations of muscular, lean, athletic, and strong bodies. We do not know how many men are adopting these images as their ideal standard, comparing themselves with this standard, and then feeling that they do not measure up. However, we do know that the body image, self-esteem and other self-concepts of Indigenous men are very negative and they are more negative than those of European men [7,8]. Moreover, an increasing number of Indigenous Australian men are either severely underweight or overweight [25]. The problem of being underweight is significant for a large number of young men who sniff petrol [31,32]; petrol sniffing is used by young men "to achieve power over their bodies" and then "their thin bodies symbolise their marginalised place" [[25], p. 164]. On the other hand, the increasing rates of obesity among Indigenous men is another significant problem, and in part attributable to the adoption of the unhealthy "Westernized" lifestyles, as discussed above [9]. Therefore, a better understanding of how Indigenous men view and care for their bodies is critical for them to improve their health and wellbeing.

### Acculturation

One key sociocultural variable to consider is acculturation. Acculturation refers to changes in identity, attitudes, values, and behaviours that accompany an individual's movement from their original or 'heritage' culture towards a new and different mainstream culture [33]. In most research into acculturation, the focus is on how immigrant groups adapt to their host country's cultural ways [34]. In the case of colonized first nation's peoples it can be used to refer to how these groups have adapted to an imposed superculture. Acculturation has been shown to be important in the development of physical and emotional health among other cultural groups [35]. However, to the authors' knowledge, no study has specifically examined the effects of acculturation on Indigenous Australian men's physical and emotional health. Although diverse characteristics of an individual are thought to be affected by acculturation (all acculturation measures assess cultural identification across numerous life domains, i.e. family, relationships, leisure, work and body image), the process of acculturation is typically conceptualized and measured as a coherent and uniform shift in all these characteristics (identification responses are combined across domains). We doubt, however, that acculturation operates in such a uniform manner. Certainly, cultural shifts in particular characteristics and within particular domains are likely to be more relevant to Indigenous men's health risk behaviours than others. Therefore, it is important to consider these different domains.

Acculturation can be measured both unidimensionally and bidimensionally. Unidimensional models of acculturation suggest that movement towards a mainstream culture necessitates the disappearance of distinctive values or perspectives related to heritage culture [36]. Alternatively, bidimensional models conceive acculturation as a process in which identification with the mainstream culture and the heritage culture are free to vary independently [37]. Given the complex and dynamic nature of Aboriginal men's cross-cultural experiences, and the challenges to Indigenous cultures that have occurred in Australia, it appears necessary to adopt a bidimensional view of acculturation and consider identification with traditional culture separately from the imposed Western culture. This will enable the determination of whether a closer identification with traditional culture is a protective factor for Indigenous men, as has been found in previous research with other (immigrant) cultural groups [38,39,18]. Given the changes brought about by the adoption of 'Westernized' lifestyles, it may also be that a closer identification with the dominant mainstream culture is associated with a more negative body image and more unhealthy patterns of eating and exercise. However, this hypothesis awaits further investigation.

### Loss of traditional roles

In addition to acculturation, another crucial factor to consider is the loss of traditional masculine roles that have resulted from colonization. This change has been well documented in the literature, and is often attributed to the breakdown of the kinship system and its related social rules as a result of interference in families and communities by the state and churches [40]. Currently, Indigenous men are viewed as fearful, confused, ashamed, angry, and lost [[41], p. 3]. These are "not qualities we could describe as ideally male or masculine" [[41], p. 3]. Moreover, "Aboriginal men no longer see themselves as the warrior, providers for the families or role models in their own community" [[41], p. 4]. Rather Indigenous men see themselves as "welfare dependents with no voice and, if suffering from mental illness no real future" [[41], p. 4]. Similarly, Adams [[42], p. 70] has argued that "Aboriginal men have lost the roles that generated prestige and self-esteem (or relational self-esteem); they have lost the ability to achieve and excel in an area beyond the family or household".

The loss of men's traditional masculine roles is likely for many men to contribute to a sense of helplessness and hopelessness or more specifically, a pessimistic explanatory style [43,44], and this is turn may be perpetuating men's unhealthy behavioural practices: "Persons who suffer rejection, discrimination, ostracism and alienation; or shaming and hostility within their families or communities; and perceive themselves as powerless victims, are likely to become self-destructive" and this can lead to a range of "unhealthy or risky behaviours, such as poor diet and little exercise" [[45], p. 45].

In order to counteract the loss of traditional masculine roles, it is crucial that we find ways to promote new roles that Indigenous men can value. One such means is via athleticism and sport. The sporting context is one of the main forums that Western males have for demonstrating the various aspects of masculinity that are closely aligned with the pursuit of fitness and muscularity. These include athletic strength and superiority, competitiveness, toughness, endurance, leadership, status, power, and authority [46]. Increasingly, sport is also becoming a strong and positive influence for many Indigenous men. For example, the importance of sport in the lives of young Indigenous men has been linked to the company of peers and positive interactions with older men. For example, McCoy et al. [[25], p. 25]: proposed "young men need older men to watch over them and 'grow them up', older men also need those opportunities to care for, protect and teach those who are younger, and in turn be cared for by younger men". Other positives associated with sports, and football in particular, include the importance of 'joining in together' [[25], p. 174] and providing "an energetic social and geographical 'space' for exercise, discussion and male group activity." [[47], p. 82]. Importantly too, "the skills that are needed within the (Australian Rules) football arena are very similar to those that men have developed and learned over many years as they hunted in the desert" [[25], p. 182].

### Familial and community relationships

Promoting and valuing intergenerational and interpersonal relationships is also important for men's health. As articulated by McCoy [25], "holding" encapsulates the strong emphasis that Indigenous cultures place on intergenerational and interpersonal relationships, that is, caring and being responsible, assuming responsibility, teaching and providing company for younger people. This is clearly seen in many of the positive interactions among Indigenous men: "men travelling together; older men guiding and watching over younger men, men learning, engaging and competing in the company of other men" [[25], p. 144]. These all provide opportunities for men to integrate "the physical, social and spiritual dimensions of health and life", and thus promote "confidence, strength and dignity" [[25], p. 144]. A more developed understanding of ways in which these familial and community relationships influence men's health and health behaviours could improve the ability to design and implement health promotion strategies that effectively incorporate these factors, and thus represents another potential avenue of investigation.

In summary, the above discussion indicates a range of factors that may influence the health attitudes and behaviours of Indigenous men in Australia, and contribute to their poor health, or protect them from poor health. There is, however, a need for an increased understanding of these factors and how they may interact. This understanding must also necessarily be informed from the perspective of Indigenous men themselves. The remainder of this paper will describe the aims and expected outcomes, research framework, recruitment protocol, design, and data analytic techniques of a study designed to explore these factors and further develop our understanding of how to improve the health of Indigenous Australian men.

### Aims and expected outcomes

The overall aim of this study is to examine factors related to eating and physical activity among young Indigenous Australian men, and their attitudes and behaviours relating to their body, diet, and physical activity. By closely working with men and the communities in which they live, their notions of health, the body, well-being, and other aspects of their "life-world" can be better understood [48]. Given that Indigenous Australians are a highly migratory and heterogeneous community [49], we will work with young men from urban, rural and remote locations. There also appears to be different health problems and risk factors in these contrasting environments [50]. These different factors have yet to be evaluated systematically over varying locations and contexts, however, and it may be the case that recommendations and strategies that are effective in one setting may not work in another. Therefore, a comprehensive assessment of both risk and protective factors will be targeted over a variety of locations to advance theory development in the field.

We will aim to use the findings to identify strategic points of collaboration and action; and promote health enhancing strategies. These strategies will be developed and implemented with the Indigenous community in the collaborative manner. The expected outcome of this study is a better understanding of young Indigenous Australian men's views of health and well-being, and the sociocultural factors that contribute to their relatively poor physical and emotional health. Knowledge of these factors may then be used to develop more effective prevention strategies that promote healthy eating and positive patterns of physical activity.

## Methods and design

### Research framework

The study will be conducted using a participatory action research (PAR) framework [51]. PAR involves critical reflection and action that "aims to improve the health and reduce health inequities through involving people who, in turn, take actions to improve their own health" [[51], p. 854]. This approach values the knowledge of members of the target community, and attempts to view problems from their perspective. As such, this framework appears particularly suitable to adopt when working with this population [52,13]. A PAR framework recognises that researchers have certain technical expertise and that community members have particular knowledge of their community's needs and perspectives. Therefore, both researchers and participants contribute unique strengths and share responsibilities [17]. Further to this, researchers aim to build on the strengths, resources, and relationships that already exist within communities [53]. The importance of using this framework has been voiced by Aboriginal elders; health care workers; other researchers; and politicians [45,13]. It is also embedded in the ethical guidelines developed by the Australian National Health and Medical Research Council for working with Indigenous communities [54].

In accordance with a PAR framework, young Indigenous men will be recruited for consultation, and to directly assist with data collection. To develop the research questions, leaders in the Indigenous community will be consulted and worked with as cultural consultants and advisors to the study. Advisory panels will be set up in three locations (Broome, regional Victoria, and metropolitan Melbourne). Meetings will be held with these advisory panels at crucial stages of the study in order to inform all aspects of the research. In addition, Indigenous men will be employed as researchers in each of the three research sites described below. We will work collaboratively with these men to develop the interview protocol, recruit participants, work with men and their families, collect, analyse and interpret data, and find appropriate and sensitive ways of disseminating the findings for each stage of research. Through this approach, information gained from the study will be shaped, researched and analysed in partnership with Indigenous men living in these communities.

### Participant groups and recruitment

The term Indigenous Australian refers to a heterogeneous group of people [49]. Thus, the perspectives, attitudes, and behaviours of Indigenous men are likely to vary from one location to another. One of the major pitfalls of research in this area is that many previous studies have attempted to determine a strategy for Indigenous men without being sensitive to the substantial differences in the needs of men living in different locations and their circumstances. For health promotion strategies to be effective, it is essential that they focus on the specific needs and protective factors within individual communities. In order to ensure that our participants reflect the diversity of Indigenous Australians, we will recruit from three locations: the Broome area of the Kimberly region in far north Western Australia, regional Victoria, and metropolitan Melbourne, in the south east of Australia. These three locations represent urban, regional, and rural contexts, and may give rise to unique and relevant issues related to geographical location, contact with non-Indigenous people and culture, local customs and culture, population density etc. We will work within these communities to access samples of men aged 18-35 years.

A non-probability sampling technique that involves dimensional and snowball sampling will be used. The relevant dimensions in this study are Indigenous Australian ethnicity, age, and location. Community leaders will be asked permission to contact men within our target age group. Our Indigenous researchers, who will help develop and then be trained in our interview schedule, will then approach potential participants to be engaged in an interview. Subsequent participants will be identified by these Indigenous researchers, and through snowballing. In snowball sampling, initial participants are asked to suggest subsequent informants who would be in a position to contribute information. Snowball sampling therefore relies on the insider knowledge of subjects [55] and is a useful approach when there is difficulty in identifying or accessing members of a population, such as when there is a clandestine group [56].

### Study design

This study will be comprised of three stages. The first two stages will involve qualitative data collection involving group and individual interviews with young Indigenous Australian men (ages 18-35 years). The third stage will involve the organisation and facilitation of community events designed to transfer new knowledge back to communities, promote health-enhancing strategies, and build the capacity of communities to take future action.

### Stage one

The objectives of the first stage of the project will be to scope the factors identified in the research literature and determine which health issues are of importance among Indigenous men in each location. To achieve this, focus groups will be conducted with 10 men in the age group of 18-35 years from each of the three locations. The main areas that will be examined are outlined in Table 1. Given the expected difference between life experiences of men from urban, regional and remote areas, the initial content will be primarily determined by men from the advisory panels in each location. The advisory panels and focus groups will also be used to determine the relevance and cultural appropriateness of the interview content, as well as obtaining suggestions on additional content that may be added. If necessary, further focus groups will be conducted to clarify any of the health-related or cultural themes that emerge from the data. In order to promote a reciprocal and open dialogue with our participants, we will include extensive engagement with the local communities [53].

**Table 1:** Aims of the three stages of study

Stage 1 - Focus Groups
• Clarify areas of concern around physical activity, diet and body image;
• Examine the importance of these concerns for the Indigenous men, and the extent to which they are concerned about unhealthy practices;
• Identify factors related to these concerns (e.g., cultural identity, level of acculturation; pessimistic vs. optimistic explanatory styles); and
• Observe healthy and unhealthy practices, and differences between the men.

Stage 2 - Individual Interviews
• Determine the meaning and importance that Indigenous Australian men attach to their health and body;
• Understand the role of risk and protective factors in determining eating and physical activity patterns; • Find ways of promoting healthier eating and physical activity patterns; and
• Examine how each of the above differs in the three locations.

Stage 3 - Action and dissemination of information
• Identify strategic points of action; ways to promote health enhancing strategies; ways to transfer knowledge back to communities; and determine future action and collaboration that is needed
• Organise and facilitate two community events in each location
• Assist and support Indigenous men and local Indigenous researchers to run these events
• Assist local Indigenous men and Indigenous researchers to develop their research skills, further disseminate information, and apply for funding to continue with their specific targeted goals

### Stage two

The second stage of the project will aim to build on stage one and further explore the identified themes and factors (see Table 1 for a description of the broader aims). Semi-structured interviews will be used, as this method can provide an in-depth assessment of these factors [57]. In particular, we will use the interviews to elicit the ways in which individuals perceive and interpret the issues we are targeting and why they respond in the manner that they do [58]. As there are no validated and culturally sensitive instruments for this cultural group [29], these interviews will also help to identify any additional factors that we have overlooked, and allow us to probe answers in more depth than is possible using surveys and experimental methods.

We will first conduct individual interviews with 20 young men aged 18-35 from each of the three locations. We will then analyse these interviews and feedback these preliminary findings to the advisory panels. This interactive process is essential in order to identify missing or poorly defined issues, additional nuances, and further practical and relevant issues pertaining to each location. After using this iterative and reflective process, we will complete an additional 20 interviews from each of the three locations. The final sample size of 40 men from each location has been selected as the number required to ensure we elicit a diverse and comprehensive array of themes [59].

The content of the interview schedule used in stage two will be based on:

1. A review of the relevant literature;

2. Findings from stage one;

3. Meetings with our advisory panels and local community organisations; and

4. Background interviews and surveys the research team has previously completed with Indigenous men [60-62,7] and other cultural groups [38,39,63].

The interview schedule will likely include questions that assess men's (a) eating attitudes and behaviours; (b) type and frequency of physical activities; (c) attitudes to health and the body; (d) body change strategies currently used, used in the last 12 months and planned for the future; (e) the factors influencing men's health (i.e. identification with cultural traditions, relationship with mainstream culture, masculinity, identification with sport; intergenerational and interpersonal relationships; explanatory styles); and (f) men's acculturative experiences (i.e. culture clash, expectations to conform, conflicting cultural values).

### Stage three

The findings from stages one and two will be used to (a) identify strategic points of action; (b) develop health enhancing strategies; (c) find ways of transferring the new knowledge back to the communities; and (d) determine what future action and collaboration need to be taken. Following this, two community events will be organised and facilitated in each location to transfer these findings to the community and to promote better health practices. These events will be run by Indigenous men from each community, and our local Indigenous researchers. However, the research team and advisory groups will continue to provide support and input as needed.

The research team will also assist the Indigenous men and Indigenous researchers to develop their research skills, and disseminate the findings to both their local communities and the broader academic community, and to apply for additional funding to continue with their specific targeted goals. Importantly, and in line with the PAR framework, the researched will then become the researchers [53]. Moreover, it must be noted that outcomes, targets and goals are likely to differ in each of the three locations, depending on the issues raised in the earlier stages.

### Data analysis

The interview data will be analysed in three stages. We will firstly code the transcripts into NVivo 9 qualitative data analysis software [64] according to text/sections that relate to a specific idea and/or interview questions. N9 will then be used to locate all text units on each topic. Secondly, the research team will work individually and then collaboratively using a modified grounded theory approach [58,65] and inductive thematic analysis [66] to identify and interpret the main themes. Further input will be obtained from our meetings with men and their families, and our advisory panels in each location.

Lastly, inbuilt in our approach are several methods to establish the trustworthiness of our data [67]. These include checks to ensure the authenticity, quality and the consistency of our interpretations grounded in the data: prolonged engagement (the need for sufficient time and interaction to establish rapport, trust and purpose), triangulation (discussions with the men's families), peer debriefing (our advisory panels); and collaboration and member checking (research questions, procedures, and analyses will be co-constructed between the research team and the participants of the study). We will also take into account researcher reflexivity, as "researchers cannot be separated from the research process and must understand his or her position, as it informs his or her interpretations" [[67], p. 38].

### Consent and ethics

All participating interviewees will be provided with a plain language statement and will provide written informed consent to participate in interviews. Participating interviewees will be offered gift vouchers to compensate them for their time. The project has been approved by the Deakin University Human Research Ethics Committee (DU-HREC 2009-182), and will be approved by the relevant organisations and community elders in each location.

## Discussion

Improving indigenous health presents a significant challenge for the Australian government and service providers. The former Prime Minister, Kevin Rudd, stated on March 20, 2008, in the "Close the Gap" Indigenous Health Equality Summit Statement of Intent: "We are committed to working towards ensuring Aboriginal and Torres Strait Islander peoples have *access to health services that are equal in standard to those enjoyed by other Australians*" [italics added].^2 ^While there are a variety of structural factors that are associated with this gap (e.g., inadequate housing; lack of education and employment opportunities) and these may be addressed through government policy initiatives, an understanding of lifestyle factors and the associated beliefs underlying these is an essential foundation element of programs that are designed to improve the health of indigenous people. The development and implementation of programs that are devised without this knowledge and on the basis of Western understanding alone are unlikely to be successful.

Our project aims to develop a greater understanding of the lifestyle and associated health beliefs for young adult Indigenous Australian men, an under-resourced and sometimes forgotten group in research and service areas. We will establish the attitudinal and acculturation factors associated with health protective behaviours and health risk behaviours, and understand how these vary according to urban, regional, and rural locations. This understanding, to be developed in consultation with Indigenous communities, will then be available for utilisation by those who develop health promotion, health prevention and applied health services for Indigenous communities, thereby contributing to the reduction in the gap between life expectancy of Indigenous and non-Indigenous people. Conducted within a PAR framework, this project is designed to work with men in a range of settings to collaboratively build capacity to address this critical issue that is central to the future well-being of young Indigenous men in Australia.

## Endnotes

^1^Tjurunga are objects of religious significance for Central Australian Indigenous people. They denote sacred stone or wooden objects possessed by private or group owners together with the legends, chants, and ceremonies associated with them.

^2^Available at http://www.hreoc.gov.au/social_justice/health/statement_intent.html

## Competing interests

The authors declare that they have no competing interests.

## Authors' contributions

LAR, DM, MPM, and AM conceived the project and secured the project funding. They will manage the project. DJH and MT will work on the project. DJH produced the first draft of this paper from the research protocol which has been reviewed by all authors. All authors have read and approved the final manuscript.

## Pre-publication history

The pre-publication history for this paper can be accessed here:

http://www.biomedcentral.com/1471-2458/12/28/prepub

## References

[B1] Hunter E Staying tuned to developments Indigenous health: reflections on a decade of change Australian Psychiatry 2003 11 418 423 10.1046/j.1440-1665.2003.02030.x

[B2] Hunter E Disadvantage and discontent: A review of issues relevant to the mental health of rural and remote Indigenous Australians Aust J Rural Heal 2007 15 88 93 10.1111/j.1440-1584.2007.00869.x 17441816

[B3] Australian Bureau of Statistics Experimental Life Tables for Aboriginal and Torres Strait Islander Australians, 2005-2007 2009 Canberra

[B4] Vos T Barker B Stanley L Lopez A The burden of disease and injury in Aboriginal and Torres Strait Islander Peoples Centre for Burden of Disease and Cost-Effectiveness, School of Population Health, The University of Queensland; 2007 http://www.lowitja.org.au/files/crcah_docs/Indigenous-BoD-Report.pdf21844603

[B5] Trewin D Madden R The health and welfare of Australia's Aboriginal and Torres Strait Islander peoples 2005 Australian Bureau of Statistics: Canberra22227224

[B6] McCabe MP Ricciardelli LA Mellor D Ball K Media influences on body image and disordered eating among indigenous adolescent Australians Adolescence 2005 40 115 127 15861621

[B7] Mellor D McCabe M Ricciardelli L Ball K Body image importance and body dissatisfaction among Indigenous Australian adolescents Body Image 2004 1 289 297 10.1016/j.bodyim.2004.05.003 18089160

[B8] Ricciardelli LA McCabe MP Ball K Mellor D Sociocultural influences on body image concerns and body change strategies among Indigenous and non-Indigenous Australian adolescent girls and boys Sex Roles 2004 51 731 741 10.1007/s11199-004-0722-1

[B9] Gracey MS Nutrition-related disorders in Indigenous Australians: how things have changed Med J Aust 2007 186 15 17 17229025 10.5694/j.1326-5377.2007.tb00779.x

[B10] Lee AJ O'Dea K Mathews JD Apparent dietary intake in remote Aboriginal communities Aust J Public Health 1994 18 190 197 7948337 10.1111/j.1753-6405.1994.tb00224.x

[B11] Leonard D Beilin R Moran M Whichway kaikai blo umi? Food and nutrition in the Torres Strait Aust J Public Health 1995 19 589 595 8616199 10.1111/j.1753-6405.1995.tb00463.x

[B12] Australian Bureau of Statistics The Health and Welfare of Australia's Aboriginal and Torres Strait Islander Peoples. Canberra 200822227224

[B13] Reilly R Doyle J Rowley K Koori community-directed health promotion in the Goulburn Valley Aust Community Psychologist 2007 19 39 46

[B14] McDermott RA Commentary: Closing the health gap for Indigenous Australians--will better counting mean better services and investment in the social production of health? Int J Epidemiol 2009 38 477 479 19155281 10.1093/ije/dyn369

[B15] Vos T Barker B Begg S Stanley L Lopez AD Burden of disease and injury in Aboriginal and Torres Strait Islander People: the Indigenous health gap Int J Epidemiol 2009 38 470 477 19047078 10.1093/ije/dyn240

[B16] Palmer A Men's health- is that good enough? The Chronicle 2007 10 22 23

[B17] Pyett P Working together to reduce health inequalities: Reflections on a collaborative approach participatory approach to health research Aust New Zeal J Public Health 2002 26 332 335 10.1111/j.1467-842X.2002.tb00180.x 12233953

[B18] Reilly RE Doyle J Bretherton D Rowley KG Harvey JL Briggs P Charles S Calleja J Patten R Atkinson V Identifying psychosocial mediators of health amongst Indigenous Australians for the Heart Health Project Ethn Heal 2008 13 351 373 10.1080/13557850801903046 18701994

[B19] Ogden J Kindleth Health psychology: A textbook 2011 New York: Open University Press

[B20] Caltabiano ML Byrne DG Sarafino EP Health Psychology: Biopsychosocial Interactions 2007 Queensland: John Wiley & Sons

[B21] McDonald H East Kimberly concepts of health and illness: A contribution to intercultural health programs in northern Australia Aust Aboriginal Stud 2006 2 86 97

[B22] Rosado RQ Consciousness in Action: Toward an Integral Psychology of Liberation and Transformation 2007 England: Ile Publications

[B23] Anderson IF Baum F Bentley M Beyond Bandaids: Exploring the Underlying Social Determinants of Aboriginal Health. Proceedings of the Social Determinants of Aboriginal Health Workshop: July 2004; Darwin 2007

[B24] Anderson I Grbich C Aboriginal well-being Health in Australia: sociological concepts and issues 2006 Sydney: Prentice Hall 57 87

[B25] McCoy BF Kanyirninpa: Health, masculinity and wellbeing of desert Aboriginal men Phd thesis 2004 University of Melbourne

[B26] McCabe MP Ricciardelli LA James T A longitudinal study of body change strategies of fitness center attendees Eating behaviours 2007 8 492 496 10.1016/j.eatbeh.2007.01.004 17950938

[B27] Mellor D McCabe MP Ricciardelli L Merino ME Body dissatisfaction and body change behaviours in Chile: the role of sociocultural factors Body Image 2008 5 205 215 10.1016/j.bodyim.2008.01.004 18463011

[B28] Frederick DA Forbes GB Grigorian KE Jarcho JM The UCLA body project I: gender and ethnic differences in self-objectification and body satisfaction among 2,206 undergraduates Sex Roles 2007 57 317 327 10.1007/s11199-007-9251-z

[B29] Ricciardelli LA McCabe MP Williams RJ Thompson JK The role of ethnicity and culture in body image and disordered eating among males Clin Psychol Rev 2007 27 582 606 10.1016/j.cpr.2007.01.016 17341436

[B30] Thompson JK Carfi G The Muscular Ideal: Psychological, Social, and Medical Perspectives 2007 Washington D.C.: American Psychological Association

[B31] Submission to the Senate Inquiry into Petrol Sniffing and Substance Abuse in Central Australia http://www.aph.gov.au/Senate/committee/clac_ctte/petrol_sniffing_substance_abuse08/submissions/sub14.pdf

[B32] Brady M Torzillo P Petrol sniffing down the track Med J Aust 1994 160 176 177 8309386

[B33] Berry JW Phinney JS Sam DL Vedder P Immigrant youth: Acculturation, identify, and adaptation Applied Psychology: An International Review 2006 55 303 332 10.1111/j.1464-0597.2006.00256.x

[B34] Schwartz SJ Unger JB Zamboagna BL Szapocznik J Rethinking the concept of acculturation: implications for theory and research Am Psychol 2010 65 237 251 20455618 10.1037/a0019330 PMC3700543

[B35] Koneru VK Acculturation and mental health: Current findings and recommendations for future research Applied and Preventative Psychology 2007 12 76 96 10.1016/j.appsy.2007.07.016

[B36] Gordon MM Assimilation in American life 1964 New York: Oxford University Press

[B37] Ryder AG Alden LE Paulhus DL Is acculturation unidimensional or bidimensional? A head-to-head comparison in the prediction of personality, self-identify, and adjustment J Personal Soc Psychol 2000 79 49 65 10.1037//0022-3514.79.1.49 10909877

[B38] Mussap AJ Acculturation, body image, and eating behaviour in Muslin-Australian women Ment Health Relig Cult 2009 15 1 7 10.1016/j.healthplace.2008.08.008 18952486

[B39] Ricciardelli LA McCabe MP Mavoa H Fotu K Goundar R Schutlz J Waqa G Swinburn BA The pursuit of muscularity among adolescent boys in Fiji and Tonga Body Image 2007 4 361 371 10.1016/j.bodyim.2007.07.001 18089282

[B40] Human Rights and Equal Opportunity Commission Bringing them home: Report of the National Inquiry into the Separation of Aboriginal and Torres Strait Islander Children from their Families [Commissioner: Ronald Wilson]. Sydney 1997

[B41] Akbar R Masculinity and mental health: an Aboriginal mental health workers perspective http://www.ethicsalnutrition.com.au/menshealth/akbar.htm

[B42] Adams M Raising the profile of Aboriginal and Torres Strait Islander men's health: An Indigenous perspective Aust Aborig Stud 2006 2 68 74

[B43] Peterson C Park N Gross JJ Explanatory style and emotion regulation Handbook of Emotional Regulation 2007 New York: Guildford Press 159 179

[B44] Seligman MEP Learned Optimism 1990 New York: Knof

[B45] Lowe HJ Spry FM Living male: journeys of Aboriginal and Torres Strait Islander males towards better health and well-being http://www.health.nt.gov.au/health/comm_health/mens_health/pdf/living_male_discussion_paper.pdf

[B46] Ricciardelli LA McCabe MP Ridge D The construction of the adolescent male body through sport J Heal Psychol 2006 11 577 587 10.1177/1359105306065018 16769737

[B47] McCoy BF 'If we come together our health will be happy': Aboriginal men seeking ways to better health Aust Aborig Stud 2006 2 75 85

[B48] Stringer E Genat WJ: Action Research in Health 2004 New Jersey: Pearson Education

[B49] Foster D Mitchell J Ulrik J Williams R Population and mobility in the town camps of Alice Springs: report prepared by Tangentyere Council Research Unit http://www.tangentyere.org.au/publications/research_reports/DKCRC-Report-9-Population-and-Mobility-in-the-town-camps-of-Alice-Springs.pdf

[B50] Scrimgeour D Town or country: Which is best for Australia's Indigenous peoples? Med J Aust 2007 186 532 533 17516903 10.5694/j.1326-5377.2007.tb01030.x

[B51] Baum F MacDougall C Smith D Participatory action research J Epidemiol Community Health 2006 60 854 857 10.1136/jech.2004.028662 16973531 PMC2566051

[B52] Esler DM Participatory action research in indigenous health Aust Family Physician 2008 37 457 459 18523701

[B53] Israel BA Eng E Schulz AJ Parker EA Israel BA, Eng E, Schulz A J, Parker E A Introduction to methods in community-based participatory research for health Methods in community based participatory research for health 2005 San Francisco: Jossey-Bass 3 26

[B54] National Health and Medical Research Council Values and Ethics: Guidelines for Ethical Conduct in Aboriginal and Torres Strait Islander Health Research 2003 http://www.nhmrc.gov.au/_files_nhmrc/<!-- publications/attachments/e52.pdf

[B55] Minichiello V Aroni R Timewell E Alexander L In-depth interviewing 1995 2 Melbourne: Longman

[B56] Robson C Real World Research: A Resource for Social Scientists and Practitioners-Researchers 1993 Oxford: Blackwell

[B57] Matsudaira T Measures of psychological acculturation: a review Transcultural Psychiatry 2006 43 462 487 10.1177/1363461506066989 17090628

[B58] Strauss A Corbin J Basics of qualitative research: Grounded theory procedures and techniques 1990 United States: Sage

[B59] Shank G Qualitative Research: A Personal Skills Approach 2005 New Jersey: Merril Prentice Hall

[B60] Mellor D Contemporary racism in Australia: the experiences of Aborigines Personality and Social Psychology Bulletin 2003 29 474 486 10.1177/0146167202250914 15273002

[B61] Mellor D Responses to racism: A taxonomy of coping styles used by Aboriginal Australians Am J Orthopsychiatry 2004 74 56 71 14769109 10.1037/0002-9432.74.1.56

[B62] Mellor D Bretherton D Firth L Aboriginal and non-Aboriginal Australia: The dilemma of forgiveness in reconciliation Peace and Conflict: J Peace Psychology 2007 13 9 34

[B63] Mussap AJ Masculine gender role stress and pursuit of masculinity Int J Men's Health 2008 7 72 89 10.3149/jmh.0701.72

[B64] NVivo qualitative data analysis software; QSR International Pty Ltd. Version 7 2006

[B65] Ridge DT Plummer DC Peasley D Remaking the masculine self and coping in the liminal world of the gay 'scene' Cult Heal Sex 2006 8 501 514 10.1080/13691050600879524 17050383

[B66] Layder D New strategies in social research 1993 Cambridge: Polity

[B67] Yeh CJ Qualitative analysis and interpretation in counseling psychology: Strategies for best practice J Couns Psychol 2008 35 34 48

